# Access Rate to the Emergency Department for Venous Thromboembolism in Relationship with Coarse and Fine Particulate Matter Air Pollution

**DOI:** 10.1371/journal.pone.0034831

**Published:** 2012-04-11

**Authors:** Nicola Martinelli, Domenico Girelli, Davide Cigolini, Marco Sandri, Giorgio Ricci, Giampaolo Rocca, Oliviero Olivieri

**Affiliations:** 1 Section of Internal Medicine, Department of Medicine, University of Verona, Verona, Italy; 2 Emergency Department, Hospital of Verona, Verona, Italy; Leiden University Medical Center, Netherlands

## Abstract

Particulate matter (PM) air pollution has been associated with cardiovascular and respiratory disease. Recent studies have proposed also a link with venous thromboembolism (VTE) risk. This study was aimed to evaluate the possible influence of air pollution-related changes on the daily flux of patients referring to the Emergency Department (ED) for VTE, dissecting the different effects of coarse and fine PM. From July 1^st^, 2007, to June 30^th^, 2009, data about ED accesses for VTE and about daily concentrations of PM air pollution in Verona district (Italy) were collected. Coarse PM (PM_10-2.5_) was calculated by subtracting the finest PM_2.5_ from the whole PM_10_. During the index period a total of 302 accesses for VTE were observed (135 males and 167 females; mean age 68.3±16.7 years). In multiple regression models adjusted for other atmospheric parameters PM_10-2.5_, but not PM_2.5_, concentrations were positively correlated with VTE (beta-coefficient = 0.237; P = 0.020). During the days with high levels of PM_10-2.5_ (≥75^th^ percentile) there was an increased risk of ED accesses for VTE (OR 1.69 with 95%CI 1.13–2.53). By analysing days of exposure using distributed lag non-linear models, the increase of VTE risk was limited to PM_10-2.5_ peaks in the short-term period. Consistently with these results, in another cohort of subjects without active thrombosis (n = 102) an inverse correlation between PM_10-2.5_ and prothrombin time was found (R = −0.247; P = 0.012). Our results suggest that short-time exposure to high concentrations of PM_10-2.5_ may favour an increased rate of ED accesses for VTE through the induction of a prothrombotic state.

## Introduction

In the last decade, air pollution has been shown to affect not only respiratory but also cardiovascular morbidity and mortality [1]. Since 2004 the American Heart Association (AHA) recognized that exposure to fine particulate matter (PM) air pollution, i.e. air-dispersed particle with aerodynamic diameter less than 10 µm (PM_10_), is associated with an increased risk for cardiovascular events, particularly myocardial infarction (MI), stroke, arrhythmia, and heart failure [2]. Importantly, exacerbation within hours to days of exposure in susceptible individuals was claimed as important as higher long-term average PM levels, both these factors being potentially relevant in reducing life expectancy [1]–[4].

The pathogenic properties of PM_10_are thought to be influenced by their size, leading to subdivision in 2 groups: a coarse component with aerodynamic diameter between 2.5 and 10 µm (PM_10-2.5_) and a finest component with aerodynamic diameter less than 2.5 µm (PM_2.5_). The two components have different sources and composition. Indeed, while PM_2.5_ particles result mainly from combustion of fossil fuels from a variety of activities (e.g. traffic and industry), PM_10-2.5_ particles are associated with non-combustion surface or fugitive releases by a variety of human (e.g agriculture) and natural (e.g. erosion) activities [5]. Moreover, PM_10-2.5_ particles deposit preferentially in the upper and larger airways, while the PM_2.5_ particles may reach the smallest airways and alveoli, where the finest component (ultrafine particles <0.1 µm) can spread even into the systemic circulation throughout the alveolar-capillary wall [6]. Originally, in two landmark cohort-based mortality studies, the Harvard Six Cities [7] and the ACS studies [8], PM_10-2.5_ was not related to mortality, while PM_2.5_ was associated with mortality for both cardiovascular and pulmonary causes. On the basis of similar observations and results, PM_2.5_ has been considered as the true or, at least, the main culprit of the adverse effects of PM air pollution on the human health [1].

In very recent years, PM air pollution has been also associated with changes in the global coagulation function, suggesting an activation of the hemostatic system and an unbalanced hypercoagulability even after short-term exposure [3], [4], [9]. In addition, exposure to the air pollution seems to significantly affect the risk of venous thromboembolism (TVE) with an approximately linear exposure-response relationship over the PM range [10]–[12]. If this is the case, the amount of individuals referring to the hospital for VTE may vary accordingly to ambient PM concentrations even on relatively short periods of time.

The aims of the present study were therefore to evaluate possible influence of air pollution-related changes on the daily flux of patients referring to Emergency Department (ED) for VTE, dissecting the potentially different effects of coarse and fine PM.

## Results

**Figure 1:**
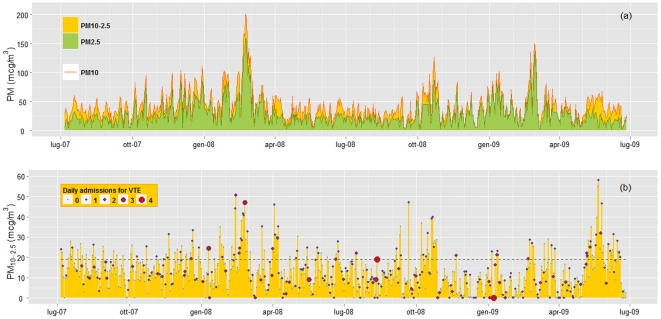
Seasonal trend of total, fine, and coarse particulate matter (PM_10_, PM_2.5_, and PM_10-2.5_) concentrations during the study period. In Figure 1A, PM_10_ levels are represented by the orange line and the area under the curve is divided in the 2 components, PM_2.5_ represented by the green area and PM_10-2.5_ represented by the ochre yellow area. In Figure 1B, the seasonal trend of PM_10-2.5_ concentrations is separately represented and related with data of daily admissions for venous thromboembolism (VTE). The dashed line represents the PM_10-2.5_ 75^th^ percentile, at 19 µg/m^3^.

During the index period (n = 640 days), 302 patients were recognized to be affected by VTE (135 males and 167 females; mean age 68.3±16.7 years). For each day, data of both PM_10_ and PM_2.5_ (and therefore also of PM_10-2.5_) were available. Figure 1 shows the seasonal trend of PM concentration during the study period. As expected, PM_10_ and PM_2.5_ concentrations were higher during the cool seasons (Figure 1A), while no clear-cut trend was found for PM_10-2.5_ concentration (Figure 1B).

At univariate analysis, PM_10-2.5_ concentration showed a significant correlation with VTE (beta coefficient = 0.163; *P* = 0.039 – Table 1), while no significant association was found for either PM_10_ (beta coefficient = 0.167; *P* = 0.064) or PM_2.5_ (beta coefficient = 0.091; *P* = 0.221).

**Table 1:** Correlation of coarse particulate matter (PM_10-2.5_) pollution with Emergency Department daily admissions for venous thromboembolism by unadjusted and multiple adjusted Poisson regression.

PM_10-2.5_	Unadjusted		Adjusted			
			Model 1†		Model 2‡	
	Beta-coefficient	*P*	Beta-coefficient	*P*	Beta-coefficient	*P*
**Venous thromboembolism**	0.163	0.039	0.246	0.003	0.237	0.020

† : adjusted for atmospheric variables (i.e. mean temperature, humidity, and air pressure).

‡ : adjusted for atmospheric variables and PM_2.5_.

Considering the strong correlation among PM data, including PM_10-2.5_, and atmospheric parameters, a specific adjustment was performed. PM_10-2.5_ concentration remained significantly associated with ED daily admissions for VTE after adjustment for atmospheric parameters (beta coefficient = 0.151, *P* = 0.002 – Table 1), as well as after the inclusion in the regression model of PM_2.5_ data (beta coefficient = 0.237; *P* = 0.020, respectively – Table 1).

**Figure 2:**
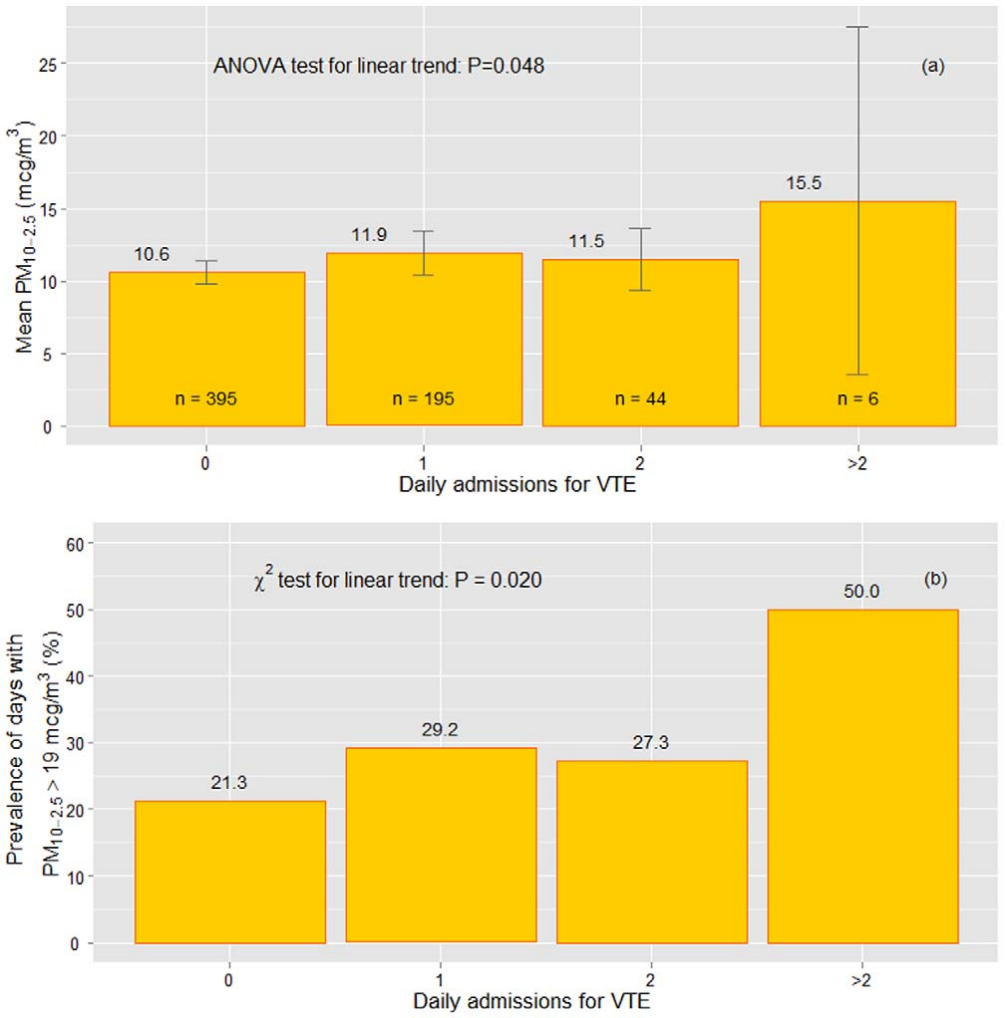
Coarse particulate matter (PM_10-2.5_) and daily admissions to the Emergency Department for venous thromboembolism (VTE). Data are presented as mean level of PM_10-2.5_ concentration (Figure 2A) and prevalence of days with high PM_10-2.5_ concentration, defined as higher than the 75^th^ percentile – 19 mcg/m^3^ (Figure 2B), according to the number of daily admissions to the Emergency Department for VTE.

Subsequent analyses showed that mean levels of PM_10-2.5_, as well as the proportion of days with high concentration of PM_10-2.5_ (defined as greater than the 75^th^ percentile, i.e. 19 µg/m^3^), progressively raised by increasing the number of ED daily admissions because of VTE (Figure 2). Interestingly, there was no sex or age difference between VTE patients during days with high or low PM_10-2.5_concentration (data not shown). Considering the daily hospital referral for VTE as a dichotomic variable (yes/no), the days characterized by higher concentration of PM_2.5-10_, i.e. ≥19 µg/m^3^, showed an increased probability (by more than 50%) to observe at least one VTE access than the days with PM_2.5-10_<19 µg/m^3^ (OR 1.69 with 95%CI 1.13–2.53 after adjustment for atmospheric parameters and PM_2.5_ data).

**Figure 3:**
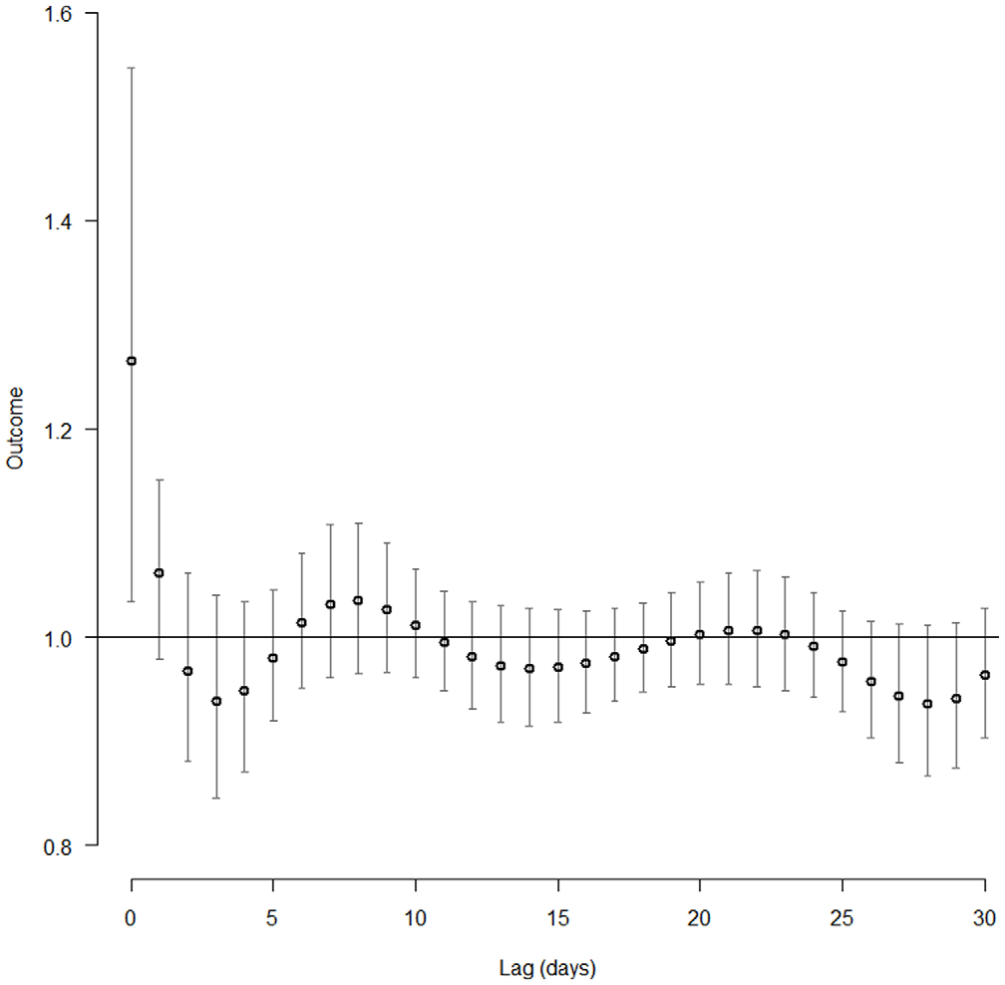
Association between daily hospital referral for venous thromboembolism (VTE) and coarse particulate matter (PM_10-2.5_) concentration at different time-lags. The association was estimated by using distributed lag non-linear models with VTE risk as outcome and time-lags expressed as the number of previous days. Only the current-day (lag 0) PM_2.5-10_ levels presented a significant association with VTE risk.

The association between daily hospital referral for VTE and PM_2.5-10_ concentrations was investigated also at different time-lags by using distributed lag non-linear models (DLNM). In such analysis the increase of VTE risk was limited to short-time exposure. Indeed, only the current-day (lag 0) PM_2.5-10_ levels presented a significant association with VTE, while considering the previous days no significant association was found (Figure 3). Moreover, in order to evaluate the stability of the estimated effects, we fitted a series of DLNM models with different configurations of parameters which showed that the reported effects remained substantially unchanged under model perturbations (data not shown).

Finally, on the basis of such results, we further investigated the associations of PM concentrations and coagulation times, both PT and aPTT. In a subgroup of study population – admitted for mild respiratory symptoms without active thrombosis and without warfarin therapy (n = 102)– an inverse correlation between PM_10-2.5_ and PT was detected, while no significant associations were found for PM_2.5_ (Table 2). Noteworthy, the correlation between PM_10-2.5_ and PT remained significant also in a linear regression model adjusted for sex, age, and the other atmospheric variables (standardized beta-coefficient −0.251; *P* = 0.035).

**Table 2:** Pearson's correlations of particulate matter (PM) concentrations, considered as a whole (PM_10_) or subdivided in the finest (PM_2.5_) and the coarse component (PM_10-2.5_), with activated partial thromboplastin time (aPTT) and prothrombin time (PT).*

		aPTT	PT
**PM_10_**	**R**	−0.119	−0.187
	***P***	0.274	0.060
**PM_2.5_**	**R**	−0.089	−0.129
	***P***	0.416	0.198
**PM_10-2.5_**	**R**	−0.112	−0.247
	***P***	0.303	0.012

* : This analysis was performed in a subgroup of patients admitted to Emergency Department for mild respiratory symptoms, without active thrombosis and not taking anticoagulant therapy, for whom data about coagulation times were available (n = 102).

## Discussion

The main result of the present study was the association between 24-h levels of coarse PM_10-2.5_ and referral rate for VTE independently from age, gender, and the other atmospheric parameters. Such association appeared to be limited to short-time exposure. Moreover, consistently with this result, there was an inverse correlation between PM_10-2.5_ levels and PT values, with shorter PT during the periods characterized by higher coarse PM air pollution.

Exposure to air pollution is well known to be associated with adverse effects on health. As regards to cardiovascular health, particulate air pollution increases morbidity and mortality due to atherothrombotic events [13]. Moreover, recent preliminary findings suggest an association of particulate air pollution also with VTE [9]–[12]. Particulate air pollution has been linked with several pathological mechanisms (reviewed in [1] and [13]) from endothelial dysfunction to prothrombotic diathesis [14]–[21]. The majority of these associations has been ascribed to the fine PM_2.5_, while the coarse particles have received less attention and emphasis.

However, things may be more complex than they appear. Indeed, also coarse PM generates oxidative stress [22], has cytotoxic properties [23] and stimulates cytokine production [24]. Many experimental findings have demonstrated that coarse PM may exert specific and characteristic toxic effects as finest PM, so that more than 10 years ago Wilson and Su proposed that “fine and coarse particulate are separate classes of pollutants and should be measured separately in research and epidemiological studies” [25].

The association between coarse PM and hospital referral for VTE implies some aspects of novelty. The first observation that particulate air pollution can enhance venous thrombosis risk was done in 2008 by Baccarelli and colleagues, who demonstrated that the exposure to PM_10_ over a long period (1 year) increased the risk of VTE [10]. A recent surveillance study in a Chilean population confirmed these data, emphasizing the effect of the finest component PM_2.5_ [11]. Moreover, in another study the risk of VTE was increased for subjects living near a major traffic road, a recognized major determinant of exposure to particulate pollution, compared with those living farther away and the increase in VTE risk was approximately linear over the observed distance range [26]. On the other hand, these results on VTE risk were not replicated by two recent prospective cohort studies [27], [28], indicating the need of further studies addressing such issue. Nonetheless, the biological plausibility of the association between particulate air pollution and VTE was supported by the observation of an inverse correlation between PM_10_ and PT, thus suggesting that air pollution-related changes favour a prothrombotic condition in the global coagulation balance [9]. PM has been associated also with transient increases in plasma viscosity, acute-phase reactants (including fibrinogen), and D-dimer [29], [30], as well as with reduced release of tissue plasminogen activator, impairing endogenous fibrinolysis [31]. Remarkably, inhalation of diluted diesel exhaust – an efficacious model for simulation of PM exposure – has been found to increase thrombus formation within 2 hours from exposure, supporting the hypothesis that also short-term exposure to PM at near-ambient levels may favour prothrombotic diathesis [12]. Noteworthy, it is not clear so far whether such changes in haemostatic balance are due to direct interactions of (circulating) PM or secondary to systemic inflammatory response (triggered by pulmonary stress).

Our results are substantially consistent with these previous works on PM-induced VTE risk, but with some remarkable differences. First, while the previous reports investigated a long-term effect of PM exposure, our results on hospital referrals point towards a direct association between PM exposure and VTE risk in the short-term period. Noteworthy, in our analysis long-term exposure for more consecutive days did not result in a more increased VTE risk. The latter result may appear unexpected at a first glance, considering that venous thrombosis is usually a process that takes time to develop and to manifest itself clinically. Nonetheless, given that acute peaks of PM exposure contributes to systemic inflammation and hypercoagulability, a link between short-term PM peaks and VTE events may be even more biologically plausible [9], [13].Considering VTE as a multifactorial disease with different steps of progression from initial and asymptomatic phases to late and clinically evident manifestations, we are tempting to speculate that peaks of coarse PM exposure may act as short-term triggers of acute VTE clinical manifestations requiring hospital referral.

Second, the most intriguing result of our analysis was that the coarse and not the finest component of PM was associated with VTE risk. PM_2.5_ and ultrafine particles, on the basis of their capacity to spread into blood circulation throughout the alveolar-capillary wall, were so far considered in experimental models as the most likely culprits influencing haemostatic balance. However, population studies have not clearly defined so far which PM components actually influence VTE. Our findings may contribute to reduce such uncertainty, providing evidence in favour of a role of coarse component PM_10-2.5_, rather than fine PM_2.5_. In our study PM_10_ tended to be associated with VTE, thus our data do not contrast substantially with the previous reports [9], [10] but extend their observations to the specific fraction PM_10-2.5_. Baccarelli et al. showed an inverse correlation between PM_10_ and PT, while they founded no association between aPTT and air pollutant level [9]. Thus, they proposed that air pollutants might alter blood coagulation through the induction of tissue factor (TF). Noteworthy, a recent study demonstrated that, although PM-driven long lasting thrombogenic effects were predominantly mediated via formation of activated FXII, PM promoted its early procoagulant actions mostly through the TF-driven extrinsic pathway [32]. Consistently with this hypothesis TF expression is enhanced during systemic inflammatory response, as well as in macrophages exposed to PM [33]. Experimental evidence supports a pro-oxidative and inflammatory role also for coarse PM [21]–[23], even much stronger than that of fine PM. On this basis, we speculate that short-term exposure to PM_10-2.5_may trigger a systemic inflammatory response through pulmonary stress, which in turn may activate blood coagulation through TF induction. Further studies are undoubtedly needed to address in depth such hypothesis.

Our study has some important limitations. First, the diagnosis classification in ED did not allow a complete characterization of the clinical phenotypes. In particular, we cannot discriminate between idiopathic or secondary VTE events. Moreover, we cannot adjust our analysis for other confounding factors, like socio-economic class. Coarse PM was calculated indirectly by subtracting PM_2.5_ from PM_10_, so that PM_10-2.5_ measurement may be affected by two measurement errors rather than just one, and ambient air pollution was used as surrogate for personal exposure. However, this approximation is considered to determine only a modest underestimation of PM effects [34]. Certainly, the biological significance of assumptions made on an arithmetic difference should be corroborated by further studies, but the consistency of PM_10-2.5_ association with both PT and VTE was impressive. Another limitation is represented by the lack of assessment of other potential air pollutants, including carbon monoxide, sulphur dioxide, nitrogen oxides, and ozone. On the other hand, one strength of our study is the full adjustment for all the common atmospheric parameters, like temperature, humidity, and pressure. Noteworthy, a recent study supports the presence of seasonal and monthly variability of VTE, to whom PM air pollution may contribute, with a significantly higher risk in winter (absolute increase of risk of about 12%), particularly in January (absolute increase of risk of about 20%) [35].

In summary, our analysis shows that coarse PM air pollution may be a significant predictor of ED admissions for VTE in a urban area in northern Italy, in particular by considering peaks of PM_10-2.5_ exposure in the short-term period. Our results support the claim that not only fine but also coarse PM may have substantial implications on human health [5], [25]. The identification of the specific PM fraction/s responsible for increased VTE risk may be useful in view of future large-scale preventive strategies against environmental air pollution. Indeed, although the individual risk of VTE triggered by PM is relatively small, the public health burden could be dramatically larger, owing to the massive exposure of the populations to this risk [36]. Moreover, as hypothesized by Mannucci, if “TVE episodes that we might call unprovoked only because none of the established risk factors are identified are perhaps provoked by air pollution” [37], then information on the local rate of daily PM should be usefully released to ED physicians facing with patients referring for possible VTE.

## Materials and Methods

From July 1^st^, 2007 to June 30^th^, 2009, all subjects who were examined in Emergency Unit of the University Hospital of Verona for medical (not surgical) problems were initially considered. Of them (n = 18,841 subjects), according to the software “FirstAid” used to classify the referring patients and on the basis of days with the availability of both PM_10_and PM_2.5_data on the admission date (n = 640 days), were subsequently selected 302 cases who were recognized to be affected by VTE (coded according to the International Classification of Diseases(ICD) as ICD-10 I26 and I82). In particular VTE was diagnosed based on evidence from objective methods such as D-Dimer increase associated with radiological demonstration of VTE obtained by means of either compression ultrasonography or lung computed tomographic angiography.

To further investigate the potential influence of PM on thrombophilic diathesis we selected a subgroup of subjects without active thrombosis, within a group of patients admitted for mild and aspecific respiratory symptoms, and for whom data of coagulation times were available (n = 102). In such group the correlations between PM air pollution and coagulation times, both prothrombin time (PT) and activated partial thromboplastin time (APTT), were analysed.

For this epidemiological study only data from the public and anonymous registry about clinical activity of the Emergency Unit of the University Hospital of Verona were used. Therefore, specific approval by the Ethic Committee of our institution (Azienda Ospedaliera Universitaria Integrata, Verona) was not needed, nor an informed consent was obtained from all the subjects who were examined in Emergency Unit of the University Hospital of Verona from July 1^st^, 2007 to June 30^th^, 2009.

### Air pollution data

Recordings of air pollution data, measured from July 1^st^, 2007 to June 30^th^, 2009, were obtained from the Regional Environmental Protection Agency (ARPAV). The records were by permanent monitors and provided data on air pollution (24-hours averages) of the Verona district; they resulted by standardized quality control procedures for the measurement of the concentration of the different pollution agents. Data about other atmospheric parameters, like temperature, barometric pressure and humidity, were also provided.

The analyzers used by ARPAV for bothPM_10_and PM_2.5_were based on a filtering pre-treatment of the air sample, aspirated by an appropriate pump system, which was then evaluated by a gravimetric method, as otherwise specified in ARPAV website [38].

The PM_10-2.5_value was obtained by the difference between the measured values of PM_10_ and PM_2.5_.

### Statistical analysis

All the statistical calculations were performed with SPSS 17.0 statistical package (SPSS Inc., Chicago, IL). Because of the skewed distribution, PM values were log-transformed and geometric means with 95% confidence intervals (95%CI) were reported.

Quantitative data were assessed using the Student's t-test or by analysis of variance (ANOVA), with polynomial contrasts for linear trend when indicated. Correlations between quantitative variables were assessed using both Pearson's correlation test and linear regression models. Qualitative data were analyzed with the χ^2^-test and with χ^2^ for linear trend when indicated. Odds ratios (OR) with 95%CI were estimated by multiple logistic regression models. In order to dissect for possible different correlations between the number of ED admissions for any group of considered disease and PM_10_, PM_2.5_, and PM _10-2.5_ values were analysed by means of log-linear model of Poisson regression, at first univariate, then adjusted for all the other atmospheric parameters (temperature, barometric pressure, and humidity).A value of *P*<0.05 was considered statistically significant.

The association between hospital referral for VTE and PM_10-2.5_concentrations at different lags was investigated using distributed lag non-linear models (DLNM) [39]. The DLNM used in the analysis was fitted through a generalized linear model with quasi-Poisson family and a canonical log-link. The lagged effect of PM_10-2.5_up to 30 days of lag was modelled by a quadratic B-spline. The concentrations of PM_10-2.5_were not centred in order to describe the effect versus a reference value of 0 mcg/m^3^. Such model was also adjusted for atmospheric parameters, i.e. temperature, atmospheric pressure, and humidity. Finally, in order to evaluate the stability of the estimated effects, we fitted a series of DLNM with different configurations of bases in the predictor-lag spaces, different values of the maximum lag and different subsets of the three atmospheric parameters. DLNM estimation was performed with software R, version 2.13.1 [40], using R package dlnm, version 1.5.2 [41].
